# Direct and indirect vitamin A supplementation strategies result in different plasma and tissue retinol kinetics in neonatal rats

**DOI:** 10.1194/jlr.M067165

**Published:** 2016-08

**Authors:** Libo Tan, Amanda E. Babbs, Michael H. Green, A. Catharine Ross

**Affiliations:** Department of Nutritional Sciences* Pennsylvania State University, University Park, PA 16802; Huck Institutes for the Life Sciences,§ Pennsylvania State University, University Park, PA 16802; Cardiometabolic Disease, † Merck Research Laboratories, Boston, MA 02115

**Keywords:** neonate, maternal dietary vitamin A, compartmental model, WinSAAM

## Abstract

Many questions remain regarding vitamin A (VA) supplementation of infants. Herein we compared direct oral VA supplementation of the neonate and indirect treatment through maternal dietary VA (M-VA) treatment on VA status and kinetics in neonatal rats. Treatments included direct VA combined with retinoic acid (RA) [D-VARA; VA (6 mg/kg) + 10% RA, given orally to neonates on postnatal day (P)2 and P3] and indirect VA supplementation through increased M-VA, compared with each other and oil-treated neonates. [^3^H]retinol was administered orally to all neonates on P4. Plasma and tissue [^3^H]retinol kinetics were determined from 1 h to 14 days post-dosing. D-VARA versus placebo dramatically increased liver and lung retinol, but only in the first 8–10 days. In M-VA neonates, liver and lung VA increased progressively throughout the study. Compartmental modeling of plasma [^3^H]retinol showed that both D-VARA and indirect M-VA reduced retinol recycling between plasma and tissues. Compartmental models of individual tissues predicted that D-VARA stimulated the uptake of VA in chylomicrons to extrahepatic tissues, especially intestine, while the uptake was not observed in M-VA neonates. In conclusion, indirect maternal supplementation had a greater sustained effect than D-VARA on neonatal VA status, while also differentially affecting plasma and tissue retinol kinetics.

Vitamin A (VA) is a critical micronutrient for development (1, 2), innate and adaptive immunity (3), and growth and survival. VA deficiency in infants and children increases the risk of mortality and infectious diseases (3–5). The demonstration that VA supplementation of at-risk children 6 months to 5 years of age reduces mortality has led to broad advocacy for VA supplementation by the World Health Organization (WHO) and other agencies (6). However, whether to recommend VA for infants <6 months of age is controversial (7), and WHO has no policy regarding <6-month-old infants.

Physiologically, newborns begin life with tissue VA levels well below those of adults. Liver retinol concentrations have been reported to be <10 μg/g liver (35 nmol/g) (8), levels which, in older children and adults, would be considered deficient. Similar low concentrations have been observed in the livers of newborn rats (9). Plasma retinol concentrations in infants are also low compared with adults, typically <1 μM and often <0.7 μM (10, 11); such concentrations would be interpreted as marginal or inadequate in older children or adults. At present, many questions remain regarding VA supplementation, including the impact of various modes of VA supplementation on the development of tissue VA reserves in the neonatal period.

In previous studies, we have used the rat as a preclinical model in which to determine the effects of VA supplementation on the kinetics and mass distribution of retinol, using methods of [^3^H]retinol tracer analysis and compartmental modeling. In one of our previous studies, we investigated VA kinetics from postnatal day 4 (P4) to P18 in neonatal rats that were either unsupplemented (oil-treated and nursed by VA-marginal dams; control group) or treated orally with a combination of VA and its active metabolite, retinoic acid (RA), at 1/10 the dose of VA (VARA). The VA mass in the dose of VARA was chosen to resemble the administration of 50,000 IU of VA, a high-dose single supplement, in human infants (12). [^3^H]retinol was included in both the oil and VARA doses as a tracer. Based on plasma and tissue tracer data, whole-body retinol kinetics were determined using model-based compartmental analysis (13–15). From the compartmental models, we obtained information on the transfer, turnover, storage, and disposal of retinol, as well as the contribution of different tissues to VA kinetics in unsupplemented and VARA-supplemented neonates. The single dose of VARA dramatically but transiently increased VA mass in neonatal plasma, liver, and lung, while it also stimulated the uptake of plasma VA into extravascular tissues (13) and markedly impacted the trafficking of VA in neonatal rats [e.g., it stimulated the uptake of newly absorbed VA carried by chylomicrons (CMs) into extrahepatic tissues (14)].

The impact of direct and indirect VA supplementation on VA status and kinetics in neonates has not been compared. In the present study, we tested two methods of VA supplementation: direct supplementation of the neonate (D-VARA), and an indirect treatment through switching the diet of the VA-marginal dams to a VA-adequate diet after parturition (M-VA). The D-VARA neonates received direct oral treatment with VARA given twice prior to the administration of an [^3^H]retinol tracer dose. Having previously observed that the effect of a single dose of VARA given simultaneously with [^3^H]retinol tracer was transient (13), we were interested, in the present study, in how D-VARA, which we predicted would build up tissue VA stores before administration of the tracer dose, would alter whole-body [^3^H]retinol kinetics. In the second group, designated M-VA, the mother’s diet was improved after the birth of her pups by feeding a VA-adequate diet throughout lactation. This strategy was based on previous studies in lactating rats in which it has been shown that dietary VA supplementation rapidly increases the concentration of retinyl esters in milk, with indirect effects on neonatal VA status (16). It is also known that significant amounts of VA are transferred from mother to offspring during lactation so that the infant can rapidly build VA stores (17). From a translational perspective, these approaches, direct and indirect supplementation, represent two very different modes of micronutrient delivery, and understanding their impacts on the neonate may help to inform the most effective means of VA supplementation in early life, with practical implications for VA supplementation in humans.

## MATERIALS AND METHODS

### Animals

Animal protocols were approved by the Pennsylvania State University Institutional Animal Care and Use Committee. Sprague-Dawley female and male rats were housed with continuous access to food and water and a 12 h light/dark cycle. After mating, the male rats were removed from cages and the females were fed an AIN-93G diet (Research Diets, New Brunswick, NJ) modified to contain a marginal level of VA, 0.35 mg retinol equivalents per kilogram diet, which was selected to reduce the transplacental transfer of VA and the concentration of VA in colostrum and milk during lactation (9). Mothers of D-VARA pups were fed VA-marginal diet throughout the study period. Mothers of M-VA pups were switched from VA-marginal to VA-adequate diet, containing 4 mg retinol equivalents per kilogram diet, on the day of birth of pups and fed this diet to the end of the study.

### Preparation of the oral dose

The amount of VA in the D-VARA oral dose [∼6 mg/kg body weight (18)] was based on the amount of VA provided as a supplement to human infants to reduce morbidity and mortality (50,000 IU/∼2.5 kg) (15), while the amount of RA in the D-VARA dose (∼0.6 mg/kg body weight) was determined in previous studies to stimulate retinol uptake and esterification in neonatal lungs (19). Retinoids for the oral dose (all-*trans* retinyl palmitate and all-*trans* RA) were purchased from Sigma-Aldrich (St. Louis, MO). Canola oil was used as the placebo and vehicle for delivering [^3^H]retinol. The oil was admixed with 11,12-[^3^H]retinol (Perkin-Elmer, Waltham, MA) so that the radioactivity in the oral dose was 0.2 μCi/μl. A volume of 0.8 μl/g body weight + 1 μl for potential loss of the dose was given to each pup. Pups in the D-VARA group were pretreated with VARA twice, on P2 and P3, prior to administration of [^3^H]retinol in oil on P4. Pups in the M-VA group were nursed by dams fed VA-adequate diet after parturition and were given the same amount of canola oil as placebo. On P4, pups in both groups received an oral dose of 11,12-[^3^H]retinol in canola oil, identical to that given to control oil-placebo-treated pups previously (13, 14). The dose was delivered directly into each pup’s mouth via a small positive displacement micropipette (Gilson, Middleton, WI). For each pup, the pipette tip used for dosing was placed in a tube containing hexane and any visible oil left on pup’s muzzle was blotted with a small “chip” of paper towel, which was extracted along with the pipette tip. Aliquots of each dose preparation were also extracted and analyzed for [^3^H]retinol to determine the dpm value for 100% of the dose. Radioactivity in the ingested dose was calculated individually for each neonate based on the total dpm in the dose minus the dpm remaining in the pipette tip and paper chip combined. Immediately after dosing, the time was recorded and pups were returned to their mothers. Pups consumed mother’s milk throughout the study.

### Kinetic study

Groups of pups (n = 3 per time per group) were euthanized at 10 times after dosing, including several early times that were included to monitor VA absorption: 1 h, 4 h, 6 h, 8 h, 15 h, 1 day, 2 days, 6 days, 11 days, and 14 days. At each time, pups were weighed and euthanized with isoflurane (Phoenix Pharmaceutical, Saint Joseph, MO). Blood was collected from the vena cava into heparinized syringes, and tissues (liver, lungs, kidneys, stomach, intestine, and the remaining carcass) were excised and rapidly frozen in liquid nitrogen. Plasma was stored at −20°C; tissue samples were stored at −80°C until analysis.

### Tissue analysis

The analytical procedure was similar to that reported previously (13, 14). Briefly, an aliquot of each plasma sample (10–60 μl) was transferred into a vial containing 4 ml of Scintiverse (Fisher Chemical) and analyzed for ^3^H content by liquid scintillation spectrometry (LSC); counting was done to achieve a 1% counting error (20). Another aliquot of plasma (25–100 μl) was saponified and analyzed for total retinol concentration by ultra-performance LC (UPLC) (13, 14). Portions of tissues were cut, weighed, and homogenized in 100% ethanol using a glass homogenizer; samples were incubated in ethanol for at least 1 h and then lipids were extracted into 6 ml hexane containing 0.1% butylated hydroxytoluene. After centrifuging the tubes, each upper hexane phase was removed into a new vial. The extraction with hexane/butylated hydroxytoluene was repeated and the two extracts were pooled. Solvent was evaporated under argon. For liver and lung tissues, a measured portion of the extract was taken for analysis of radioactivity by LSC and a portion was used for saponification and analysis of total retinol by UPLC. Previous studies have shown that, in liver and lung (18, 19, 21), >90% of total retinol is esterified and thus the values reported here for total retinol in these tissues represent mostly retinyl esters. For other tissues, all of the extract was evaporated to dryness and analyzed for ^3^H radioactivity by LSC. Before the stomach was processed, the milk curd was gently squeezed out, extracted, and analyzed for total retinol (also mostly esterified retinol) by saponification and UPLC.

### Calculation of fraction of the oral dose in plasma

The fraction of the ingested dose remaining in plasma at each sampling time for each pup was calculated as total radioactivity (dpm) in plasma divided by the radioactivity in the administered dose, as determined for each individual pup as described above. Total ^3^H dpm in plasma was calculated from the measured plasma tracer concentration at each time × estimated plasma volume, where plasma volume was calculated as pup’s body weight × 0.035 ml plasma per gram body weight. The fraction of the ingested dose remaining in a given tissue was calculated from the total dpm contained in that tissue divided by the dpm in the ingested dose for that pup.

### Compartmental modeling

Model-based compartmental analysis was applied to the tissue tracer response profiles (mean fraction of the ingested dose at each time for each group vs. time after dose administration) for neonatal rats in both groups using the Windows version of the Simulation, Analysis and Modeling software (WinSAAM) (22). Compartmental models of VA kinetics in neonatal rats developed in our previous study (13, 14) were used as the initial proposed models for the current analysis. The models are described in the Results.

Plasma and tissue tracer data were analyzed in light of the proposed models using WinSAAM until a satisfactory fit was obtained between observed and model-predicted values. Briefly, model parameters (fractional transfer coefficients; see below) were iteratively adjusted until a close fit was obtained, as judged by visual inspection of the simulated tracer data plot and by statistical analysis, including the sum of squares from weighted nonlinear regression analysis and the estimated fractional standard deviation (FSD) for each kinetic parameter. When necessary, the model structure was also adjusted. An F-statistic (23) and the Akaike Information Criterion (23) were used to statistically test whether increases in model complexity were statistically justified. Model complexity was increased only when it resulted in a significant improvement in the sum of squares, as determined by an F-statistic and reduced Akaike Information Criterion by more than 1–2 units. Once a satisfactory fit was achieved, final estimates of the fractional transfer coefficients [L(I,J)s] and their statistical uncertainties were generated by nonlinear regression analysis in WinSAAM. Parameters were considered well-identified if their estimated variability (FSD) was less than 0.5.

### Model-derived parameters

Fractional transfer coefficients [L(I,J)s] are defined as the fraction of retinol in compartment J transferred to compartment I each day. L(I,J)s are parameters that define the behavior of the system. The following parameters were calculated from model-generated L(I,J)s (24): mean transit time [t(I)] or turnover time is the mean of the distribution of times that a retinol molecule entering compartment I spends there during a single transit before leaving reversibly or irreversibly; mean residence time [T(I,J)] is the average of the distribution of times that a retinol molecule spends in compartment I before irreversibly leaving it after entering the system via compartment J; recycling number [ν(I)] is the average number of times a retinol molecule recycles through compartment I before it irreversibly exits from compartment I; fractional catabolic rate (FCR) is the fraction of the retinol pool that is utilized each day. Parameters were then compared between groups.

### Data interpretation

To interpret the effects of the D-VARA dosing and M-VA supplementation during lactation on VA status and kinetics in neonatal rats, we used data for the control group in our previous study (13, 14) as a reference. For all three groups, oil control, D-VARA, and M-VA, the tracer dose of [^3^H]retinol was administered in canola oil. Therefore tracer kinetics in the three groups are comparable, differing only because of pretreatment with D-VARA or maternal dietary M-VA supplementation.

### Statistical analysis

Data for tissue VA mass are reported as mean ± SEM. Differences among groups, *P* < 0.05, were determined by two-way ANOVA followed by a Bonferroni post test using Prism software (GraphPad, La Jolla, CA). Compartmental modeling was done using group mean data at each time [“super-pup” model (25)]. For kinetic parameters, L(I,J)s are presented with estimated FSDs. Differences in L(I,J)s between groups, *P* < 0.05, were determined by using an unpaired *t*-test. Briefly, a *t*-statistic for each L(I,J) was calculated using the equation (Value_1_ − Value_2_)/square root (SEM_1_^2^ + SEM_2_^2^), which was then applied to the *t*-table to determine the *P* value.

## RESULTS

### VA mass in plasma, liver, lungs, and milk

**Fig. 1.**
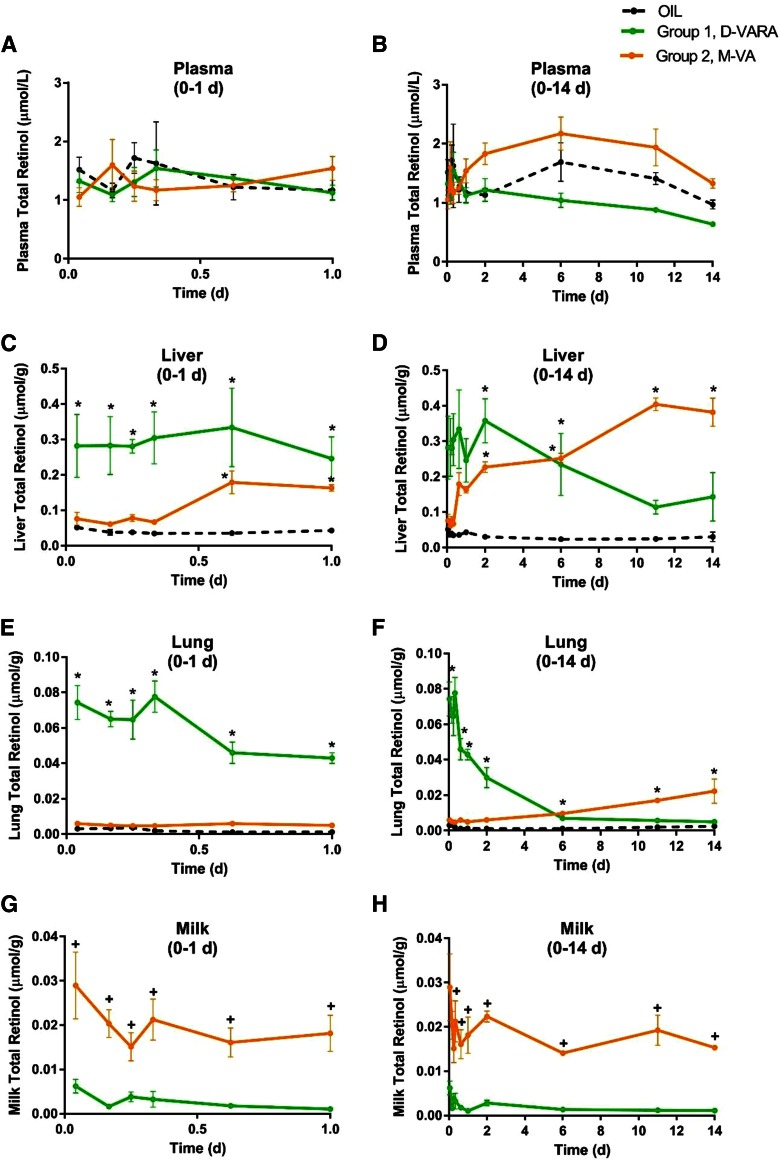
Concentrations of total retinol in plasma (A, B), liver (C, D), lung (E, F), and milk separated from neonatal stomach (G, H) versus time (days) after administration of [^3^H]retinol in oil to neonatal rats. Plots of data from the first 24 h after dose administration, expanded from panels (B), (D), and (F), are shown in panels (A), (C), and (E). Points shown are means ± SEM, n = 3 per time per group. *Significant differences between the treated groups and the control group at the same time point as indicated (*P* < 0.05). In panels (G) and (H), **^+^** indicates significant differences between the two treated groups at the same time point as indicated (*P* < 0.05).

Plasma retinol concentration in all three groups (**Fig. 1A, B**) was relatively constant (0.9–1.5 μM) throughout the study period. M-VA pups tended to have a higher plasma retinol concentration as compared with D-VARA pups from 1 day after dosing until the end of the study.

Liver total retinol (Fig. 1C, D) in the control group was less than 0.05 μmol/g at all times, consistent with values for neonates of dams fed a VA-marginal diet. Pups pretreated with D-VARA had significantly higher liver retinol concentrations in the first 6 days after administration of the tracer of [^3^H]retinol on P4 as compared with the control group, with an ∼6-fold increase at 1 h, 4 h, 6 h, 8 h, 15 h, 1 day, 2 days, and 6 days (P10) after dosing (all *P* < 0.01). However, the concentration of retinol then gradually decreased. In contrast, in M-VA pups, the liver total retinol concentration increased steadily with time until the end of the study and was significantly higher (4- to 8-fold) than that in the control group (all *P* < 0.01 at 15 h, 1 d, 2 days, 6 days, 11 days, and 14 days after dosing). Thus, whereas both the D-VARA pretreatment of the neonate and indirect M-VA treatment improved liver VA content in the neonate, compared with the VA-marginal state, the kinetics were different, with D-VARA resulting in only a transient increase in liver VA stores, while M-VA treatment resulted in a gradual steady accumulation of VA in the neonatal liver.

Treatment effects on neonatal lung total retinol (Fig. 1 E, F) resembled those for liver. D-VARA dramatically increased lung total retinol in the first 6 days after tracer administration, reaching ∼40- to 80-fold higher as compared with the control group (all *P* < 0.01 at 1 h, 4 h, 6 h, 8 h, 15 h, 1 day, 2 days, and 6 days). However, by day 11, lung retinol concentration in the D-VARA group had declined to the same level as that in the control group. M-VA supplementation gradually and more moderately increased neonatal lung total retinol concentration and the increase was significant (*P* < 0.05) at 6 days, 11 days, and 14 days after dosing, as compared with the control group.

The total retinol concentration in the ingested milk is shown in Fig. 1 G, H for the D-VARA and M-VA groups. Milk VA concentration in the M-VA group increased from the beginning to the end of the study (*P* < 0.01 at all times, as compared with the D-VARA group), and as compared with previous measurements of milk VA in neonates of dams fed a VA-marginal diet (26), which was comparable to our control group.

### Kinetics of tissue VA

**Fig. 2.**
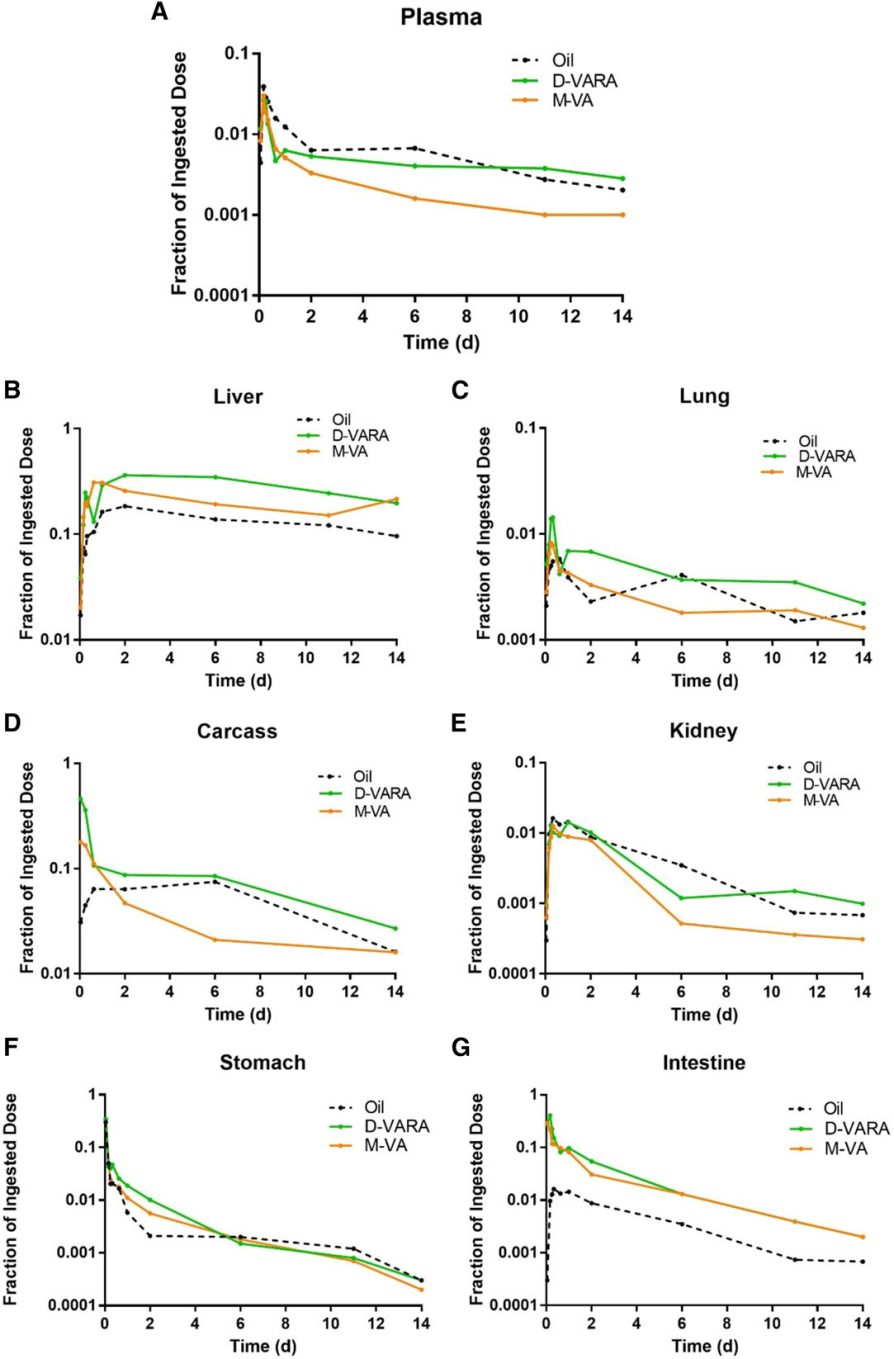
Mean observed fraction of administered dose in plasma (A), liver (B), lung (C), kidney (D), carcass (E), stomach (F), and intestine (G) versus time (days) after administration of [^3^H]retinol to neonatal rats. Each point represents the mean of n = 3 pups.

Data on the fraction of the [^3^H]retinol dose remaining in plasma, liver, lungs, stomach, intestine, kidneys, and carcass versus time after dose administration are plotted semi-logarithmically in **Fig. 2**. Radioactivity in plasma (Fig. 2A) rose quickly during the first 4 h after dose administration, with the absorption peak at 4 h, at 3.9, 2.7, and 3.0% of the administered dose in the control, D-VARA, and M-VA groups, respectively. After that, tracer rapidly disappeared from plasma, when there was a leveling off of the plasma tracer response curve, which reflects the recycling of retinol among plasma, liver, and extrahepatic tissues. Radioactivity further declined into a terminal slope, which represents the disposal of retinol. VA supplementation, whether directly as pretreatment with D-VARA or indirectly as M-VA treatment throughout the nursing period, decreased the fraction of the [^3^H]retinol dose in plasma; however, the curves differed between groups. In M-VA pups, there was a dramatic decrease in the fraction of the [^3^H]retinol dose in plasma from 6 h after dosing until the end of the study. Worthy of note is that plasma radioactivity was nearly constant in the control group from 2 to 6 days, while a constant period was not observed for either the D-VARA group or the M-VA group.

In the liver (Fig. 2B), ^3^H increased steadily until 2 days after dosing in the control and D-VARA pups, whereas it peaked in M-VA pups at 15 h after dosing. The maximum recovery of radioactivity in liver was 18, 36, and 31% in the control, D-VARA, and M-VA pups, respectively. The fraction of the ^3^H dose remaining in liver at all time points was greatly increased by both the direct and indirect modes of VA supplementation.

In the lungs (Fig. 2C), ^3^H increased within the first several hours and reached a peak 8 h after dosing in all groups. The maximum recovery of radioactivity in lung was 0.55, 1.43, and 0.82% in the control, D-VARA, and M-VA groups, respectively. After the peak, radioactivity decreased quickly and then remained relatively constant. The radioactivity in the lungs in both the D-VARA and M-VA groups declined to a level similar to that in the control group by the end of the study.

In the kidneys (Fig. 2D), ^3^H in the control group was higher than in either treatment group at most time points. The fraction of the dose was much lower in the M-VA group from 2 days after dosing until the end of the study.

The maximum recovery of the dose remaining in the carcass (Fig. 2E), mainly composed of skin, bone, muscle, brain, heart, and adipose tissue, was 11, 46, and 18% in the control, D-VARA, and M-VA groups, respectively. The fraction of the ingested ^3^H dose in carcass from 2 days to 11 days after dosing was much lower in the M-VA group compared with the control group.

In the stomach (Fig. 2F), about 35% of the tracer was found 1 h after dose administration in all groups. Tracer disappeared rapidly from the stomach in all groups, such that, at 2 days after dosing, the percentage of the administered dose in the stomach was only ∼0.2%.

For the intestine (Fig. 2G), ^3^H increased in the first several hours after dose administration in the control and D-VARA groups and, at the peak, the fraction of the administered dose reached 4.3 and 40%, respectively. This rise in the radioactivity, however, was not observed in the M-VA group, in which radioactivity was highest (29% of dose) at the earliest point and then steadily declined.

### Retinol turnover and recycling as determined by plasma tracer kinetics

**Fig. 3.**
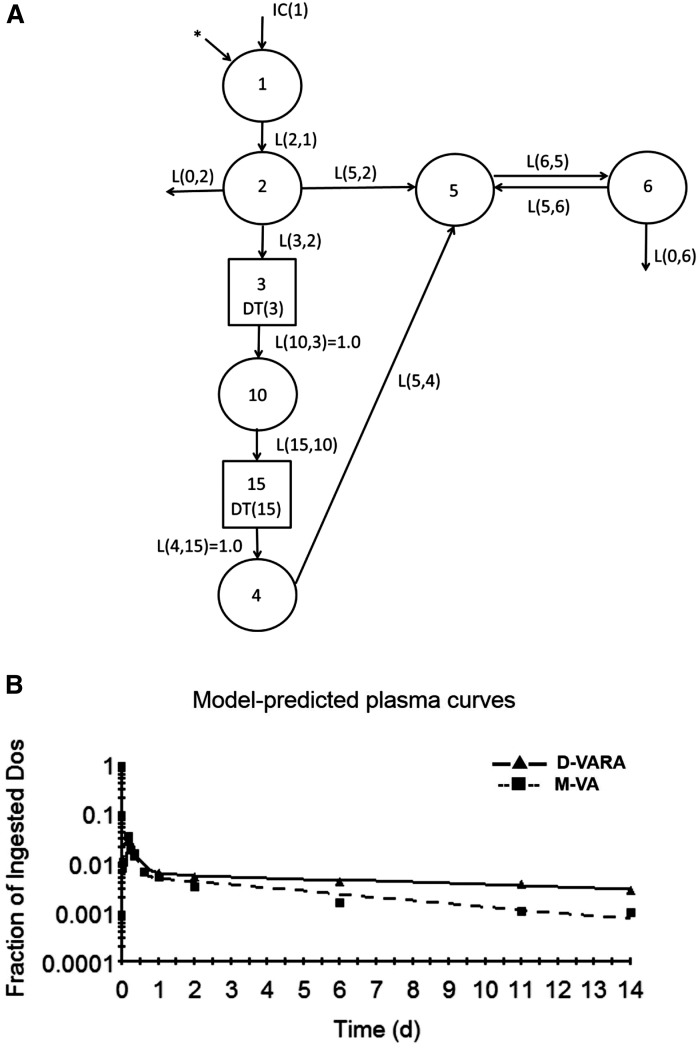
Proposed multi-compartmental model for VA metabolism as viewed from plasma space in neonatal rats after administration of [^3^H]retinol. A: Compartments are represented as circles; interconnectivities between compartments correspond to L(I,J)s, or the fraction of retinol in compartment J transferred to compartment I per day. Components 3 and 15 are delay elements. Compartments/component 1–3 represent VA digestion and absorption. Compartments/component 10, 15, and 4 represent CM metabolism, the uptake of CM remnants, and the processing of VA in extravascular tissues. Compartment 5 represents plasma retinol bound to RBP; this retinol exchanges with VA in one extravascular pool (compartment 6). The asterisk represents the site of input of [^3^H]retinol in the oral dose and is also the site of input of dietary VA. B: Mean observed (symbols) and model-predicted fraction of administered dose (lines) in plasma versus time (days) after administration of [^3^H]retinol to neonatal rats. Each point represents the mean of n = 3 pups.

A multi-compartmental model of VA kinetics as viewed from the plasma space, described in detail in (13), was developed based on the plasma tracer responses of neonates in each treatment group. The plasma view model provides information on the transfer, turnover, and disposal of retinol. The structure of our plasma view model, as previously determined for oil and VARA-treated neonates studied previously (13), is shown in **Fig. 3A**. The flow from compartment 1 (the site of input of [^3^H]retinol and dietary VA) to compartment 4 represents the processes of VA digestion, absorption, CM production, and metabolism. Delay element 3 corresponds to the time required for CM production before CMs are secreted into plasma; compartment 10 represents newly absorbed retinyl esters in plasma CMs; and delay element 15 corresponds to CM metabolism before the uptake of CM retinyl esters into liver and extrahepatic tissues, which is represented by compartment 4. After the processing of VA, protein-bound retinol, presumably on retinol-binding protein (RBP), is secreted into plasma (compartment 5). Compartment 5 represents retinol-RBP in plasma; this VA exchanges with VA in one extravascular pool (compartment 6); compartment 6 is the site of irreversible loss of VA. To obtain a good fit of the plasma kinetic data to the model for the D-VARA and M-VA groups, only changes in model parameters, but no change in the model structure, were necessary. Based on visual inspection, it is evident that the model provided a good fit to plasma tracer data for both groups (Fig. 3B).

Selected parameters derived from the plasma view model are shown in **Tables 1**, **2**. Listed in Table 1 are fractional transfer coefficients [L(I,J)s] and their estimated FSDs of the models for three groups. L(6,5), the fraction of retinol in compartment 5 (plasma retinol-RBP) that is transferred to compartment 6 (extravascular VA) each day, was significantly higher in both treatment groups than in the control group. Mean transit times, residence times, FCRs, and recycling numbers for retinol in the three groups are shown in Table 2. The calculated residence time in plasma [T(5,5); Table 2] for the control group indicates that a retinol molecule spends 2.4 days in plasma before irreversible loss. Prior treatment with D-VARA decreased the retinol residence time in plasma to 0.18 day. A retinol molecule spent only 0.06 days in plasma before it was irreversibly lost when M-VA pups were nursed by mothers switched to the VA-adequate diet at parturition. The estimated ν(5), the number of times a retinol molecule recycles through plasma, indicated that retinol recycles 144 times through plasma before irreversible loss in control neonatal rats. The D-VARA treatment and M-VA supplementation dramatically decreased the retinol recycling numbers to 17 and 5, respectively.

**TABLE 1.** Fractional transfer coefficients for D-VARA and M-VA groups compared with oil control group as predicted by the plasma view model

	Treatment		
L(I,J) (Transfer from compartment J to I)	Oil	D-VARA	M-VA
	L(I,J) values and (FSD, day^−1^)*^a^*		
L(5,4)	5.06 (0.14)	9.34 (0.09)	8.57 (0.11)
L(6,5)	60.3 (0.07)	124 (0.04)*^b^*	102 (0.03)*^b^*,*^c^*
L(5,6)	0.60 (0.10)	1.05 (0.06)*^b^*	0.59 (0.06)*^c^*
L(0,6), before day 8	0.004 (0.79)	0.05 (0.08)*^b^*	0.13 (0.04)*^b^*,*^c^*
L(0,6), after day 8	0.20 (0.06)	0.05 (0.08)*^b^*	0.13 (0.04)*^c^*

The model is shown in Fig. 3.

a Shown are model-predicted fractional transfer coefficients [L(I,J)s] or fraction of retinol in compartment J that is transferred to compartment I each day (estimated fractional SDs in parentheses).

b Significant differences (*P* < 0.05) from the oil group.

c Significant differences (*P* < 0.05) from the D-VARA group.

**TABLE 2.** Mean transit times, residence times, FCRs, and recycling numbers in neonatal rats in D-VARA and M-VA groups as predicted by the plasma-view model

	Treatment Group		
Parameters*^a^*	Oil	D-VARA	M-VA
t(5), h	0.40	0.24	0.26
t(6), days	1.64	1.14	1.03
T(5,5), days	2.40	0.18	0.06
FCR(5,5), day^−1^	0.42	5.56	16.7
FCR(5,5), day^−1^ (after day 8)	14.7	5.56	16.7
ν(5)	144	17	5

The model is shown in Fig. 3. Oil values taken from (13).

a Parameters shown are: mean transit time [t(5)], or the mean of the time that a retinol molecule spends in compartment 5 (plasma retinol-RBP) during a single transit before leaving reversibly or irreversibly; mean transit time [t(6)], or the mean of the time that a retinol molecule spends in compartment 6 (extravascular tissue retinol) during a single transit before leaving reversibly or irreversibly; mean residence time [T(5,5)], or the average of the distribution of times that a molecule of retinol spends in compartment 5 (plasma retinol-RBP) before it leaves irreversibly; FCR(5,5), or the fraction of the plasma retinol pool that is utilized each day; recycling number [ν(5)], or the average number of times a retinol molecule recycles through compartment 5 (plasma retinol-RBP) before it irreversibly exits from it.

### Contribution of different tissues to VA uptake and trafficking

**Fig. 4.**
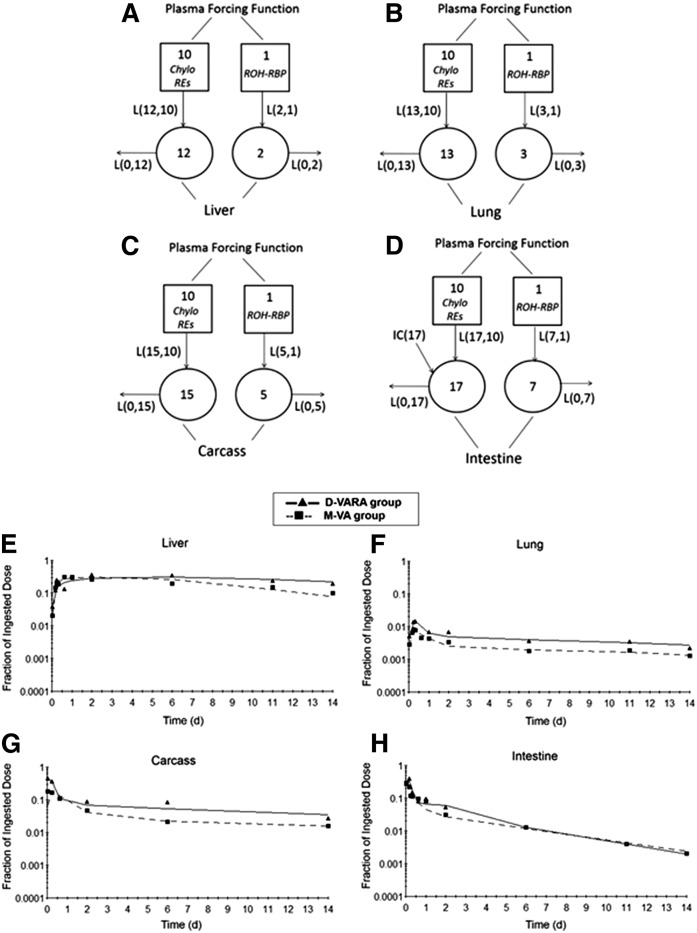
Proposed models with forcing function for VA metabolism in liver (A), lung (B), carcass (C), and intestine (D) in neonatal rats administered [^3^H]retinol based on the tissue tracer response data. Compartments are represented as circles; interconnectivities between compartments correspond to L(I,J)s, or the fraction of retinol in compartment J transferred to compartment I per day. The squares represent plasma forcing functions. Compartment 10 describes the profile of plasma tracer in CM/CM remnants retinyl ester. Compartment 1 describes the profile of plasma tracer in retinol. Compartments 12, 13, and 15 represent retinyl ester that is taken up from plasma CM/CM remnants by liver, lung, and carcass, respectively. Compartments 2, 3, 5, and 7 represent VA in liver, lung, kidney, carcass, and intestine, respectively, that exchanges with plasma retinol. Compartment 17 represents the processing of newly ingested VA in intestine and retinyl ester that are taken up from plasma CM/CM remnants by intestine. Initial condition (IC)(17) represents the newly ingested dose that enters the intestine. E–H: Mean observed (symbols) and model-predicted fraction of administered dose (lines) in liver (E), lung (F), carcass (G), and intestine (H) versus time (days) after administration of [^3^H]retinol to neonatal rats. Each point represents the mean of n = 3 pups.

The contribution of different tissues to whole-body VA kinetics and the trafficking of VA between plasma and organs can be studied through compartmental models developed for individual tissues. We developed individual tissue models by applying the “forcing function” option in WinSAAM. This approach is based on the fact that plasma is the sole source of VA to individual organs, except for stomach and intestine. As a result, individual organs can be uncoupled from the whole system and modeled individually based on the plasma tracer response profile and tracer data from the organ. Details of the development of individual tissue models with forcing function can be found in our previous publication (14). **Figure 4A–D** shows the individual models developed for four main tissues of VA metabolism: liver, lung, intestine, and carcass. Models established for liver, lung, and carcass are similar. The model for liver will be presented as an example. Compartment 12 represents retinyl ester that is taken up by the liver from plasma CM remnants (compartment 10) and compartment 2 represents liver VA that exchanges with plasma retinol (compartment 1). The tracer data for liver were assigned to compartment 12 plus compartment 2. For the intestine, because plasma is not the sole source of VA, the model includes compartment 17, which represents the processing of newly ingested VA in the intestine and compartment 7, the intestinal VA that exchanges with plasma retinol (compartment 1). To fit the intestine tracer data, an input from compartment 10 [L(17,10)] was necessary, indicating that there is some uptake of CM VA by the intestine. The fit of the proposed models to the data in both the D-VARA and M-VA groups, by criteria of the sum of squares from nonlinear regression analysis conducted by WinSAAM, can be seen by comparing the observed data (points) and the model-predicted values (lines) in different tissues (Fig. 4E–H).

The estimates of fractional transfer coefficients [L(I,J)s] and FSDs obtained from the forcing function model for the three groups are shown in **Table 3**. The value for L(12,10) indicates that fewer CM VA pools go to the liver each day in the D-VARA group and M-VA group as compared with the control group, while more CM VA pools go to the lung each day in these two treatment groups, especially in the D-VARA group, as indicated by L(13,10). L(15,10), which represents the fraction of CM VA that is taken up by carcass, was also significantly (*P* < 0.01) increased in both the D-VARA and M-VA groups compared with the control group. The fraction that is taken up by carcass was increased ∼14 times by treatment with D-VARA. Values for L(17,10), the fraction of CM VA taken up by the intestine each day, were 10.2 for the control group and 797 for the D-VARA group. In contrast, for M-VA pups, the value was 0. L(0,12) and L(0,15), which represent the fraction of the retinol tracer dose derived from CM/CM remnant that leaves the liver and carcass each day, respectively, were greatly decreased (*P* < 0.01) in the two treatment groups, especially in the M-VA group, indicating that both the D-VARA and M-VA treatments slowed the turnover of CM-derived VA in the liver and the carcass.

**TABLE 3.** Fractional transfer coefficients predicted by the forcing function model for liver, lung, carcass, and intestine

			Treatment group		
L(I,J)*^a^*	Tissues	Process Represented	Oil	D-VARA	M-VA
			*Value (d^−1^; FSD)*		
L(12,10)	Liver	CM VA to liver	168 (0.13)	90.2 (0.07)*^b^*	70.5 (0.05)*^b^*,*^c^*
L(0,12)	Liver	VA derived from CM out from liver	50.0 (0.15)	1.60 (0.84)*^b^*	0.86 (1.62)*^b^*
L(2,1)	Liver	Retinol-RBP to liver	36.9 (0.05)	27.8 (0.15)*^b^*	28.5 (0.12)*^b^*
L(0,2)	Liver	Retinol-RBP out from liver	1.20 (0.06)	0.41 (0.15)*^b^*	0.32 (0.11)*^b^*
L(13,10)	Lung	CM VA to lung	2.68 (0.04)	18.3 (0.11)*^b^*	8.70 (0.03)*^b^*,*^c^*
L(0,13)	Lung	VA derived from CM out from lung	4.07 (0.05)	20.5 (0.25)*^b^*	1.11 (0.07)*^b^*,*^c^*
L(3,1)	Lung	Retinol-RBP to lung	0.21 (0.04)	6.83 (0.11)*^b^*	0.11 (0.08)*^b^*,*^c^*
L(0,3)	Lung	Retinol-RBP out from lung	0.16 (0.04)	7.57 (0.11)*^b^*	0.10 (0.12)*^b^*,*^c^*
L(15,10)	Carcass	CM VA to carcass	89.9 (0.09)	1290 (0.06)*^b^*	229 (0.04)*^b^*,*^c^*
L(0,15)	Carcass	VA derived from CM out from carcass	14.1 (0.11)	7.88 (0.08)*^b^*	1.17 (0.06)*^b,c^*
L(5,1)	Carcass	Retinol-RBP to carcass	6.06 (0.04)	17.0 (0.15)*^b^*	1.17 (0.09)*^b^*,*^c^*
L(0,5)	Carcass	Retinol-RBP out from carcass	0.29 (0.04)	1.5 (0.16)*^b^*	0.09 (0.14)*^b^*,*^c^*
L(17,10)	Intestine	CM VA to intestine	10.2 (0.07)	797 (0.18)*^b^*	0 (0.00)*^b^*,*^c^*
L(0,17)	Intestine	VA derived from CM out from intestine	2.79 (0.09)	7.41 (0.12)*^b^*	3.95 (0.79)
L(7,1)	Intestine	Retinol-RBP to intestine	2.26 (0.04)	10 (0.13)*^b^*	10 (1.20)
L(0,7), before day 8	Intestine	Retinol-RBP out from intestine	0.29 (0.02)	0.97 (0.18)*^b^*	1.74 (1.01)
L(0,7), after day 8	Intestine	Retinol-RBP out from intestine	0.39 (0.05)	0.24 (0.04)*^b^*	1.74 (1.01)

The models are shown in Fig. 4A–D.

a The data are fractional transfer coefficients [L(I,J)s or the fraction of retinol in compartment J that is transferred to compartment I each day (estimated fractional SDs in parentheses)] predicted by the forcing function model (shown in Fig. 4), which is comprised of compartmental models developed for individual tissues with forcing function applied. L(I,10)s represent the fraction of plasma retinyl ester in CM/CM remnants that is taken up/cleared by tissues each day. L(0,I)s [L(0,12), L(0,13), L(0,15), and L(0,17)] are the fraction of retinol that leaves tissues after being processed each day that came from CM. L(I,1)s are the fraction of retinol that is transferred from plasma into tissues each day. L(0,I)s [L(0,2), L(0,3), L(0,5), and L(0,7)] represent the fraction of recycled retinol that leaves tissues each day. Initial conditions (IC)(17) are the calculated fraction of the dose at t_0_ in intestine.

b Significant differences (*P* < 0.05) from the control group.

c Significant differences (*P* < 0.05) from the D-VARA group.

As indicated by values for L(2,1), L(3,1), L(5,1), and L(7,1), the D-VARA treatment reduced the fraction of retinol-RBP that was taken up by the liver each day, as compared with the control group, while this treatment significantly (*P* < 0.05) increased the fraction taken up by the lungs, carcass, and intestine. These same fractional transfer coefficients indicate that M-VA decreased the pools of retinol-RBP going to the liver, lung, and carcass. Values for L(0,2), L(0,3), and L(0,5) indicate that D-VARA greatly decreased (*P* < 0.01) the fraction of retinol-RBP turnover out of the liver each day, but significantly increased (*P* < 0.05) the fraction of retinol-RBP turnover out of the lungs and the carcass. In M-VA pups, the lower values for L(0,2), L(0,3), and L(0,5) indicate that fewer RBP-retinol pools leave the liver, the lung, and the carcass each day. From these results, we infer that the hepatic uptake and metabolism of retinol-RBP was reduced by both treatments, and the uptake and turnover of retinol-RBP in the lung and in the carcass was accelerated by the direct treatment with D-VARA, but was slowed by the indirect M-VA supplementation.

## DISCUSSION

In this study, we investigated how VA supplementation given directly to pups versus indirectly as increased dietary VA intake provided to dams affects VA status and retinol kinetics in neonatal rats by conducting tracer kinetic experiments and model-based compartmental analysis. We found that both strategies, D-VARA and M-VA, improved the VA status of the rat pups and altered whole-body retinol kinetics, but the results differed between these two treatments, as well as from that of oil-treated unsupplemented neonates.

Neonatal rats in the control (oil) group (13, 14) and those in the D-VARA group from the current study were nursed by dams fed a VA-marginal diet to resemble the VA status of at-risk newborn infants in low-income countries where VA deficiency is still a significant public health problem, or in low birth weight infants in the US (7). The dams in the M-VA group were also fed VA-marginal diet until the pups were born and, thu,s the VA status of the dams of all of the pups was the same prior to birth. Based on total retinol measurements (Fig. 1), the control pups had a marginal to adequate plasma VA level according to the criteria used for adults (27), while their liver retinol concentration (∼0.04 μmol/g) indicated a marginal VA status, as expected. Indeed, as noted previously (13), such values would be considered deficient in older animals. Our previous study showed that a one-time supplementation with VARA, given simultaneously with the oral [^3^H]retinol dose, had a dramatic effect on increasing retinol mass in plasma, liver, and lungs (13). At day 1 after dosing, liver retinol mass started to show a significant difference as compared with that in the control group, while a significant increase in lung retinol mass was detected as early as 4 h after dosing. However, the effect was transient. By 2 days after dosing, the effect of VARA treatment on plasma retinol disappeared, and there was no apparent impact of VARA supplementation on tissue retinol concentration in the liver and lungs after 4 days. In the current study, the two-times treatment with D-VARA was given in advance of the tracer dose to build tissue retinol stores before administration of the labeled retinol, to resemble a VA-adequate status. Pups in this group already had a liver retinol concentration of 0.1 μmol/g at day 0 (P4). D-VARA dramatically increased liver and lung retinol concentration in the first 8–11 days. The liver total retinol concentration of pups in this group was 0.1–0.4 μmol/g before 11 days, so they maintained an adequate VA status for most of the study period. However, liver and lung retinol total concentrations declined gradually after 8 days and returned to a level similar to that in control pups at the end of the study. On the other hand, M-VA, which provided an adequate intake of VA to the mother beginning at and continuing throughout lactation, increased the liver and lung total retinol concentrations of the neonates progressively, from ∼0.06 μmol/g on day 0 to ∼0.4 μmol/g at the end of the study. M-VA greatly and consistently increased milk VA content, the only source of dietary VA for the pups, as was expected. From these data, we infer that high-dose D-VARA supplementation to neonatal rats has a dramatic but transient effect, while dietary M-VA supplementation to lactating mothers has a gradually increasing and sustained effect on improving VA status in the offspring.

Improving the VA status of neonates and infants could potentially save the greatest number of lives because infants are at higher risk of mortality than older children (17). However, as mentioned earlier, WHO has no policy regarding VA supplementation of infants <6 months of age. Our results indicate that the effects of high dose VA treatment directly given to neonates on improving their VA status are dramatic but transient. Previous reports on children 6–59 months of age also indicated that high dose VA supplementation had a positive but transient effect on serum retinol level (28). Repeated high-dose treatments could be considered, but would add the concern of VA toxicity. In comparison, VA supplementation of the mother during lactation seems more sustained, effective, and safe. It is known that mother’s milk is the best dietary VA source for the neonates and the infants and that the VA content of the milk is dependent on the mother’s VA status (17). As shown in our study, when the diet offered to mothers was switched from VA-marginal to VA-adequate, the VA content in the milk was rapidly and significantly increased. Thus, our study suggests that VA supplementation to lactating mothers should be an effective way to increase the VA content in the breastmilk and improve the VA status of the neonates and infants. For severe VA deficiency in neonates and infants, however, VA supplementation to lactating mothers might not be sufficiently rapid. In such cases, direct and maternal VA supplementation could be combined to benefit neonates rapidly and to provide a longer sustained improvement in VA intake.

In the current study, we explored the impacts of different VA supplementation strategies on VA metabolism and kinetics in neonates, which could potentially provide scientific basis on the understanding of the effects of these supplements on VA status. Our results indicated that VA kinetics in neonates were also dramatically and differently altered by the D-VARA dosing and M-VA supplementation. Tracer kinetic curves (Fig. 2) showed that both methods of VA supplementation stimulated the uptake of [^3^H]retinol from plasma into liver and lungs. It has been reported that VARA upregulates the mRNA expression of the gene, STRA6 (“stimulated by RA gene 6”), which is responsible for the uptake of retinol in the neonatal lung (29). This could explain the stimulation of retinol uptake by the D-VARA dosing. It has also been reported that the expression of lecithin:retinol acyltransferase, which is responsible for the esterification and storage of retinol, is higher in liver and lungs in rats with VA-adequate status compared with VA-marginal or VA-deficient rats (30). This might explain why more VA was taken up by the liver and lungs in M-VA pups, possibly for storage. Ross et al. (30) reported that the level of expression of lecithin:retinol acyl­transferase in rat liver is VA deficient < VA marginal < VA adequate < VA supplemented < RA-treated, which is consistent with our finding that the fraction of dose in neonatal liver is D-VARA treated > M-VA (mothers fed VA-adequate diet) > oil treated (mothers fed VA-marginal diet). It is worth mentioning that the fraction of dose remaining in the carcass was much lower in the M-VA group, as compared with the other groups, from 4 days to 10 days after dosing. Our previous results indicate that, when pups are under VA-marginal status, carcass (primarily composed of skin, bone, muscle, adipose tissue, brain, etc.) seems to play a more important role in retinol turnover (14). The current finding might indicate that the role of the carcass is diminished when pups have adequate VA status.

The multi-compartmental model, as viewed from the plasma space (Fig. 3A), predicted that the time that retinol spends in plasma before irreversible disposal was decreased in both treatment groups (Table 2). This finding is consistent with the prediction that the uptake and storage of VA in extrahepatic tissues are stimulated by these treatments, so that each retinol molecule spent less time in plasma. One interesting finding is that the recycling number for retinol between plasma and tissues was greatly decreased in the two treatment groups in our current study. The recycling number in the D-VARA and M-VA groups was 17 and 5, respectively, while it was 144 in the control group. The recycling number of retinol among plasma, liver, and extrahepatic tissues in adult rats is ∼12–13 (31, 32). Previously, we concluded that the more extensive recycling of retinol in pups with VA marginal status in the control group, as compared with adults, might be an adaptive mechanism to the lower VA level in pups (33). It might make retinol more available when tissue demands for retinol increase. Current results indicate that, when VA status of pups was boosted to being adequate in the D-VARA and M-VA groups, recycling was decreased to a much lower level, similar to that in adults. The molecular mechanism responsible for this finding is unknown and is worth further investigation.

The compartmental models developed for individual tissues (Fig. 4A–D) shed light on the contribution of different tissues to whole-body VA kinetics. Our previous results showed that neonatal extrahepatic tissues play an important role in clearing CM VA and that giving VARA to neonates dramatically stimulated the uptake of CM VA by the extrahepatic tissues, especially the lungs, the intestine, and remaining carcass (14). Approximately 48% of CM VA was taken up by extrahepatic tissues in control pups; whereas in VARA-treated pups, liver took up only 22% of plasma retinyl ester and 78% was taken up by extrahepatic tissues (14). The current study showed that the uptake of CM VA by the lungs, carcass, and intestine was also significantly stimulated by D-VARA (Table 3). Kinetic curves (Fig. 2) showed that the peak fraction of dose in intestine and carcass was dramatically increased by D-VARA: from 4.3% in the control group to 40% and from 11% in the control group to 46%, respectively. Thus, the VARA treatment given to neonates, no matter whether simultaneously with the tracer as in our previous study or in advance of the tracer as in the present study, dramatically stimulated the uptake of CM VA. The stimulation was also observed in the lungs and carcass of M-VA pups, but less significantly, and there was no uptake of CM VA by the intestine in those pups. These results indicate that VA supplementation and differences in VA status, which may result from the mother, can dramatically affect VA trafficking to different tissues in neonates.

Because neonates presumably have a high requirement for VA but are born with low VA status, we speculate that the uptake of CM VA by extrahepatic tissues in neonates is an adaptive mechanism to make VA more readily available for utilization by these tissues. It was previously reported that LPL is able to catalyze the hydrolysis of retinyl esters in CMs and may facilitate the uptake of retinol by these tissues in adult rats (34, 35). It is likely that LPL contributes to CM VA uptake by extrahepatic tissues in neonates as in adults. It has been shown that the activity of LPL in rat lung, brown adipose tissue, skeletal muscle, and heart muscle increased substantially during the first 24 h after birth and the activity in skeletal muscle and brown adipose tissue was highest during suckling compared with other periods of life (36). The VARA treatments in our study, especially the RA component, might stimulate the uptake of CM VA by extrahepatic tissues by increasing the expression and/or activity of LPL in these tissues. This can also explain the less significant stimulation of CM VA uptake by the lungs and carcass of pups in the M-VA group, which did not receive RA doses. In addition, for these pups with VA adequate status, the adaptive mechanism to make VA more readily available for neonatal extrahepatic tissues by increasing CM VA uptake might not be necessary.

Results for the intestine in both our previous and current studies are very interesting. As shown in Fig. 3G, a rise in radioactivity in the intestine was observed in the first several hours in the control group and D-VARA group, as observed for the other tissues except the stomach, followed by a decrease. That rise was also observed in the VARA group in our previous study (14). Because both the intestinal content and the intestinal wall were analyzed for radioactivity, the rise could not be attributed to delayed absorption. The kinetic curve for the stomach (Fig. 3F) shows that radioactivity declined rapidly and there was no delay in the emptying of the VA. A delay in stomach emptying was thus not the explanation for the rise in radioactivity in the intestine. We then considered the possibilities of enterohepatic circulation and the recycling of CM VA to the intestine to account for the rise in radioactivity when modeling the intestine tracer data. As discussed in our previous publication (14), we found that to obtain a good fit for kinetic data for the intestine, it was necessary to include uptake of CM VA, after absorption, back to the intestine. As shown for the individual tissue model (Fig. 4A–D), we used L(17,10) to represent the uptake of CM VA by the intestine. Previous results (14) showed that a single VARA treatment dramatically stimulated the uptake of CM VA by the intestine, estimated as ∼31.8% of CM VA total uptake in VARA-treated pups, compared with 4.68% in the oil-treated group. D-VARA dosing in the present study also dramatically stimulated the uptake of CM VA by the intestine (Table 3), as mentioned. It has been previously reported that RA administered either iv or as a pellet had significant trophic effects in bowel-resected rats (37). The effects of D-VARA treatments on the kinetics of retinol flux between plasma and intestinal pools might indicate a positive influence of VA on intestinal maturation. Interestingly, the rise in radioactivity in the first several hours was not observed in the M-VA group, in which radioactivity was highest at the earliest point and then steadily declined (Fig. 3G). The model predicated that L(17,10) is 0, indicating that there is no uptake of CM VA by the intestine in those neonates nursed by mothers whose diet was switched from VA-marginal to VA-adequate at the time of delivery. Future experiments focusing on the metabolism of CM VA may provide new insights into neonatal VA intestinal physiology.

In conclusion, the two methods of VA supplementation we have tested differed in their impact on neonatal tissue VA concentrations, and in the kinetics of plasma retinol turnover and its exchange with target organs. The results from the M-VA group suggest that indirect supplementation may offer advantages for a sustained impact on neonatal VA status.

## References

[1] Clagett-Dame M., and DeLuca H. F. 2002 The role of vitamin A in mammalian reproduction and embryonic development. Annu. Rev. Nutr. 22: 347–381. 12055350 10.1146/annurev.nutr.22.010402.102745E

[2] Altucci L., and Gronemeyer H. 2001 Nuclear receptors in cell life and death. Trends Endocrinol. Metab. 12: 460–468. 11701345 10.1016/s1043-2760(01)00502-1

[3] Palmer A. C. 2011 Nutritionally mediated programming of the developing immune system. Adv. Nutr. 2: 377–395. 22332080 10.3945/an.111.000570 PMC3183589

[4] Ma Y., and Ross A. C. 2005 The anti-tetanus immune response of neonatal mice is augmented by retinoic acid combined with polyriboinosinic:Polyribocytidylic acid. Proc. Natl. Acad. Sci. USA. 102: 13556–13561. 16157890 10.1073/pnas.0506438102 PMC1224655

[5] Sommer A., and West K. P. 1996. Vitamin A Deficiency: Health, Survival, and Vision. Oxford University Press, New York.

[6] UNICEF. Goal: Reduce child mortality. Accessed June 14, 2016, at http://www.unicef.org/mdg/childmortality.html.

[7] WHO. 2009. Global Prevalence of Vitamin A Deficiency in Populations at Risk 1995–2005. World Health Organization, Geneva, Switzerland.

[8] Dahro M., Gunning D., and Olson J. A. 1983 Variations in liver concentrations of iron and vitamin A as a function of age in young American children dying of the sudden infant death syndrome as well as of other causes. Int. J. Vitam. Nutr. Res. 53: 13–18. 6853055

[9] Davila M. E., Norris L., Cleary M. P., and Ross A. C. 1985 Vitamin A during lactation: Relationship of maternal diet to milk vitamin A content and to the vitamin A status of lactating rats and their pups. J. Nutr. 115: 1033–1041. 4020482 10.1093/jn/115.8.1033

[10] Hustead V. A., Gutcher G. R., Anderson S. A., and Zachman R. D. 1984 Relationship of vitamin A (retinol) status to lung disease in the preterm infant. J. Pediatr. 105: 610–615. 6481538 10.1016/s0022-3476(84)80432-1

[11] Berggren Söderlund M., Fex G. A., and Nilsson-Ehle P. 2005 Concentrations of retinoids in early pregnancy and in newborns and their mothers. Am. J. Clin. Nutr. 81: 633–636. 15755833 10.1093/ajcn/81.3.633

[12] WHO. 2011. Guideline: Vitamin A Supplementation in Infants and Children 6–59 Months of Age. World Health Organization, Geneva, Switzerland. 24575452

[13] Tan L., Wray A. E., Green M. H., and Ross A. C. 2014 Retinol kinetics in unsupplemented and vitamin A-retinoic acid supplemented neonatal rats: a preliminary model. J. Lipid Res. 55: 1077–1086. 24711633 10.1194/jlr.M045229 PMC4031939

[14] Tan L., Wray A. E., Green M. H., and Ross A. C. 2014 Compartmental modeling of whole-body vitamin A kinetics in unsupplemented and vitamin A-retinoic acid supplemented neonatal rats. J. Lipid Res. 55: 1738–1749. 24914038 10.1194/jlr.M050518 PMC4109768

[15] WHO. 2012. WHO technical consultation on vitamin A in newborn health: mechanistic studies, Geneva, Switzerland, 1–3 December 2009. World Health Organization, Geneva, Switzerland.

[16] Ross A. C., Pasatiempo A. M. G., and Green M. H. 2004 Chylomicron margination, lipolysis, and vitamin A uptake in the lactating rat mammary gland: Implications for milk retinoid content. Exp. Biol. Med. (Maywood). 229: 46–55. 14709776 10.1177/153537020422900106

[17] Stoltzfus R. J. 1994 Vitamin A deficiency in the mother-infant dyad. SCN News. 11: 25–27. 12288232

[18] Ross A. C., Ambalavanan N., Zolfaghari R., and Li N-q. 2006 Vitamin A combined with retinoic acid increases retinol uptake and lung retinyl ester formation in a synergistic manner in neonatal rats. J. Lipid Res. 47: 1844–1851. 16685080 10.1194/jlr.M600061-JLR200

[19] Ross A. C., Li N-q., and Wu L. 2006 The components of VARA, a nutrient-metabolite combination of vitamin A and retinoic acid, act efficiently together and separately to increase retinyl esters in the lungs of neonatal rats. J. Nutr. 136: 2803–2807. 17056804 10.1093/jn/136.11.2803 PMC3843131

[20] Kelley S. K., and Green M. H. 1998 Plasma retinol is a major determinant of vitamin A utilization in rats. J. Nutr. 128: 1767–1773. 9772148 10.1093/jn/128.10.1767

[21] Ross A. C., and Ambalavanan N. 2007 Retinoic acid combined with vitamin A synergizes to increase retinyl ester storage in the lungs of newborn and dexamethasone-treated neonatal rats. Neonatology. 92: 26–32. 17596734 10.1159/000100083 PMC3843127

[22] Wastney M. E., Patterson B. H., Linares O. A., Greif P. C., and Boston R. C. 1999. Investigating Biological Systems Using Modeling. Academic Press, San Diego, CA.

[23] Landaw E. M., and DiStefano J. J. 1984 Multiexponential, multicompartmental, and noncompartmental modeling. II. Data analysis and statistical considerations. Am. J. Physiol. 246: R665–R677. 6720989 10.1152/ajpregu.1984.246.5.R665

[24] Green M. H., and Green J. B. 1990 Experimental and kinetic methods for studying vitamin A dynamics in vivo. *In* Methods in Enzymology. L. Packer, editor. Academic Press, San Diego, CA. 304–317. 10.1016/0076-6879(90)90035-y 2087182

[25] Cifelli C. J., Green J. B., and Green M. H. 2005 Dietary retinoic acid alters vitamin A kinetics in both the whole body and in specific organs of rats with low vitamin A status. J. Nutr. 135: 746–752. 15795428 10.1093/jn/135.4.746

[26] Zhang Y., Wray A. E., and Ross A. C. 2012 Perinatal exposure to vitamin A differentially regulates chondrocyte growth and the expression of aggrecan and matrix metalloprotein genes in the femur of neonatal rats. J. Nutr. 142: 649–654. 22357747 10.3945/jn.111.152660 PMC3301986

[27] Pilch S. M. 1987 Analysis of vitamin A data from the Health and Nutrition Examination Surveys. J. Nutr. 117: 636–640. 3585513 10.1093/jn/117.4.636

[28] Palmer A. C., West K. P., Dalmiya N., and Schultink W. 2012 The use and interpretation of serum retinol distributions in evaluating the public health impact of vitamin A programmes. Public Health Nutr. 15: 1201–1215. 22401130 10.1017/S1368980012000560

[29] Wu L., and Ross A. C. 2010 Acidic retinoids synergize with vitamin A to enhance retinol uptake and STRA6, LRAT, and CYP26B1 expression in neonatal lung. J. Lipid Res. 51: 378–387. 19700416 10.1194/jlr.M001222 PMC2803240

[30] Ross A. C. 2003 Retinoid production and catabolism: role of diet in regulating retinol esterification and retinoic acid oxidation. J. Nutr. 133: 291S–296S. 12514312 10.1093/jn/133.1.291S

[31] Green M. H., Green J. B., and Lewis K. C. 1992 Model-based compartmental analysis of retinol kinetics in organs of rats at different levels of vitamin A status. *In* Retinoids: Progress in Research and Clinical Applications. M. A. Livrea and L. Packer, editors. Marcel Dekker, New York. 185–204.

[32] Green M. H., Uhl L., and Green J. B. 1985 A multicompartmental model of vitamin A kinetics in rats with marginal liver vitamin A stores. J. Lipid Res. 26: 806–818. 4040952

[33] Tan L., Green M. H., and Ross A. C. 2015 Vitamin A kinetics in neonatal rats vs. adult rats: comparisons from model-based compartmental analysis. J. Nutr. 145: 403–410. 25540407 10.3945/jn.114.204065 PMC4336526

[34] van Bennekum A. M., Kako Y., Weinstock P. H., Harrison E. H., Deckelbaum R. J., Goldberg I. J., and Blaner W. S. 1999 Lipoprotein lipase expression level influences tissue clearance of chylomicron retinyl ester. J. Lipid Res. 40: 565–574. 10064745

[35] Blaner W. S., Obunike J. C., Kurlandsky S. B., al-Haideri M., Piantedosi R., Deckelbaum R. J., and Goldberg I. J. 1994 Lipoprotein lipase hydrolysis of retinyl ester. Possible implications for retinoid uptake by cells. J. Biol. Chem. 269: 16559–16565. 8206972

[36] Cryer A., and Jones H. M. 1978 Developmental changes in the activity of lipoprotein lipase (clearing-factor lipase) in rat Lung, cardiac muscle, skeletal muscle and brown adipose tissue. Biochem. J. 174: 447–451. 708397 10.1042/bj1740447 PMC1185933

[37] Wang J. L., Swartz-Basile D. A., Rubin D. C., and Levin M. S. 1997 Retinoic acid stimulates early cellular proliferation in the adapting remnant rat small intestine after partial resection. J. Nutr. 127: 1297–1303. 9202083 10.1093/jn/127.7.1297

